# Modelling and Analysis of Expansion Joints’ Effect on Dynamic Performance of Railway Rigid Overhead System

**DOI:** 10.3390/s23156797

**Published:** 2023-07-29

**Authors:** Xiaohe Feng, Zeyao Hu, Shibin Gao, Fuchuan Duan, Wenping Chu, Yang Song

**Affiliations:** 1 National Rail Transit Electrification and Automation Engineering Technique Research Centre, Southwest Jiaotong University, Chengdu 610031, China; xiaohe_feng@126.com (X.F.); daxianhu@126.com (Z.H.); gao_shi_bin@126.com (S.G.); duanfc_cd@outlook.com (F.D.); 18706102509@163.com (W.C.); 2 China Railway Construction Electrification Bureau Group Rail Transit Equipment Co., Ltd., Changzhou 213179, China; 3 SWJTU-Leeds Joint School, Southwest Jiaotong University, Chengdu 610031, China

**Keywords:** overhead system, pantograph, current collection quality, railway, optimisation

## Abstract

This study focuses on developing a comprehensive model of a rigid overhead system, which includes essential components such as the suspension structure, positioning clamp, and expansion joint. The modelling approach utilizes finite element theory and beam elements to accurately represent the displacement, stiffness, and mass characteristics of the system. The models also incorporate the suspension structure and positioning line clamp, which play crucial roles in suspending and positioning the busbar. Various suspension structures and positioning line clamps are evaluated based on their dynamic characteristics. The expansion joint, responsible for connecting different anchor sections of the rigid overhead system, undergoes a detailed analysis. Different assembly scenarios, including ideal and deflected assembly conditions, are considered. To simulate the dynamic behaviour of the expansion joint, additional beams are introduced into the system model. The primary finding of the analysis is that the maximum stresses observed in the constructed expansion joint model, under different temperature conditions and normal/deflected assembly conditions, remain within the permissible stress limits of the material. This indicates a high level of safety. However, certain areas exhibit stress concentration, particularly at the sliding block B and sliding rod A positions. This stress concentration is primarily attributed to the unique assembly form of the expansion joint. To improve stress distribution and enhance service reliability, the analysis suggests optimizing the installation deflection angle and geometric design of the expansion joint. Furthermore, the concentrated mass at the expansion joint significantly impacts the current collection quality of the pantograph-overhead system. Mitigating this negative impact can be achieved by reducing the mass of the expansion joint.

## 1. Introduction

**Figure 1:**
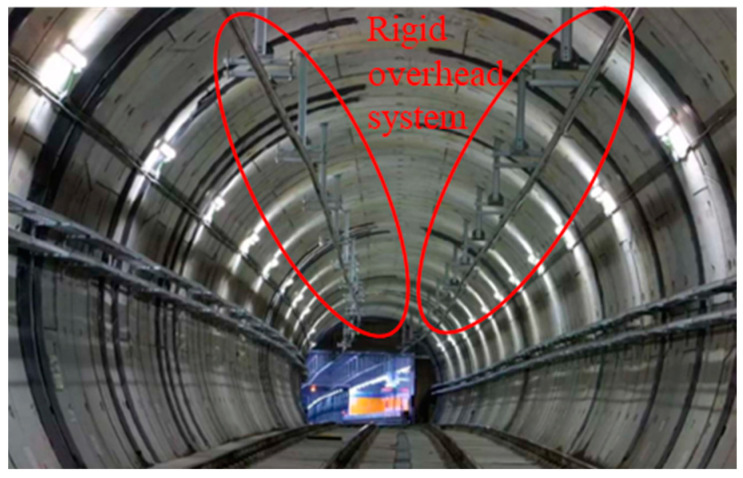
Rigid overhead system.

In electrified railways, rigid overhead systems and flexible overhead systems are two mainly used approaches for supplying electricity to the train. The main difference lies in the fact that the flexible overhead system is suspended overhead through support columns, cantilever arm systems, and tensioning cables, offering greater overall elasticity [1]. On the other hand, rigid OCS primarily relies on fixed positioning supports on tunnel walls or columns for suspending the contact wire overhead. The contact wire is clamped and secured by a busbar, and the system is characterized by high stiffness, small spans, and low clearance, as shown in Figure 1. Currently, worldwide electric locomotives primarily rely on the interaction between a moving pantograph and a stationary overhead system for power collection, enabling locomotives to acquire power and electrical supply. The dynamic contact stability of the pantograph and overhead system highly affects the safety of the power supply to the locomotives. In the case of rigid overhead systems, due to their overall rigidity, the interaction between the pantograph and the contact wire during high-speed sliding tends to be rigid, and the quality of current collection (contact force) by the pantograph is significantly affected by local irregularities in the rigid overhead system components. With the continuous development of urban rail transit around the world and the constant increase in subway operating speeds, the application of rigid overhead systems has become more widespread. The deterioration of current collection quality caused by components of the rigid overhead system and the resulting limitation on operating speeds have become more significant. Engineering application results have shown that for a rigid overhead system designed for an operating speed of 160 km/h, the vehicle operating speed needs to be reduced by 12.5% or even 25% to meet the performance requirements for the current collection by the pantograph.

The expansion of electric railway networks globally presents various technical challenges due to the complex coupling relationships among various subsystems [2,3]. One pressing issue in railway dynamics is the interaction between the pantograph and the contact wire, which directly determines the power transmission stability of the train [4]. Most previous studies have concentrated on the traditional flexible overhead system, which is the primary power supply solution for electric trains in open-air lines. Various models regarding the flexible overhead system have been extensively developed [5]. Among them, the finite element method is widely used to model the mechanical behaviour of overhead systems [6]. The measurement data [7,8] and the technical standard [9] are normally used to validate the accuracy of the numerical model. The numerical model includes complex sections of the overhead system, such as curves [10] and overlap sections [11], of which the effect on the contact force of pantograph-contact wire has been extensively analysed. The theoretical analyses of the wave propagation [12,13] and critical speeds [14,15,16] have been extensively conducted to understand the dynamics. Typically, the pantograph is modelled as a lumped mass [17] or a multibody model [18]. Various approaches have been developed for co-simulating the pantograph and overhead systems. The interaction between the pantograph and contact wire is achieved using the penalty function method [19] or the Lagrange multiplier method [20]. To accurately replicate pantograph-contact wire behaviour, hardware-in-the-loop tests have been conducted, utilizing a realistic pantograph and a mimic overhead system [21,22]. Moreover, in order to reflect complex working conditions, numerical simulations of pantograph–contact wire interaction considering overhead system defects [23], contact wire irregularities [24,25], wave disturbances [13,26], vehicle–track perturbations [27,28], and wind load [29,30,31,32] have been carried out to evaluate their effects on contact quality. With the advance of artificial intelligence, the data-driven model of the pantograph-overhead system has emerged based on neural networks [33,34,35].

There has been limited research on rigid overhead systems. One study, referenced as [36], proposes a finite element model that takes into account the large inertia moment of the conductor rail. The study analyses the dynamic performance of the pantograph-overhead system with different inertia moments of the conductor rail. In another study [37], a finite element model of rigid overhead systems using Ansys is developed. The authors highlight that the horizontal moment of the cross-sectional area of the rigid overhead contact line contributes to the fluctuation of the contact force. In [38], a model of a rigid overhead system based on elastic beam elements is presented. The study achieves a simulation of the dynamic interaction between the pantograph and the contact wire by incorporating a multibody pantograph model. Furthermore, in [39], the mechanism of wave-induced wear of the contact line under a T-shaped conductor rail is investigated. The results indicate that the unevenness of the contact line caused by construction errors has a significant impact on wear. 

The above studies contribute to the overall assessment of the rigid overhead system, its dynamic performance, contact force fluctuations, dynamic interaction between pantograph and contact wire, and the impact of construction errors on wear. However, more research is needed in modelling the key components of rigid overhead systems due to the limited extent of the existing studies. One of the important components is the expansion joint, used in rigid overhead systems to accommodate the thermal expansion and contraction of the system’s components, such as the conductor rail or contact wire. As temperature changes, these components expand or contract, and without proper allowance for this movement, it could lead to excessive stresses and damage. The design and construction of expansion joints in rigid overhead systems are crucial to ensure the safe and reliable operation of the system. In [40], a comprehensive investigation is carried out by constructing a rigid catenary-pantograph model that incorporates expansion joints. The study focuses on analysing the effects of operating speed, uplift force, and span on contact forces. Furthermore, Chover [41] examines the influence of various rigid catenary structures on the dynamic characteristics of the catenary-pantograph system, specifically considering aspects such as anchoring joints and rigid-flexible transition structures. This work aims to develop a rigid overhead system model including more detailed components, such as the suspension structure, positioning clamp, and expansion joint. Both the 3D solid model (for static analysis) and the equivalent model (for dynamic analysis) of the expansion joint are presented. Considering two working conditions, namely, normal and deflected assemblies, the stress distribution caused by the temperature variation is analysed. The optimisation approach is implemented for the stress distribution of the expansion joint. The dynamic performance of the pantograph-rigid overhead system with different configurations of expansion joints is also investigated. It is important to clarify that the objective of this paper is not to contribute to the field of computational mechanics per se but rather to employ the well-established finite element approach to investigate the effects of expansion joints on the dynamic performance of the railway rigid overhead system.

## 2. Numerical Modelling of Rigid Overhead System

In this section, the finite element approach that has been widely used in various engineering backgrounds [42,43] is adopted here to model the rigid overhead system. In the rigid overhead system, the contact wire is clamped by a busbar. According to the requirements of the People’s Republic of China Railway Industry Standard [44], the sliding load of the busbar-clamped contact wire should be greater than or equal to 103 N/m. The contact wire is tightly connected to the busbar, and they move synchronously. Therefore, the busbar and the contact wire clamped to it can be regarded as a whole. The dynamic characteristics of this combined entity can be simulated using beam elements.

**Figure 2:**
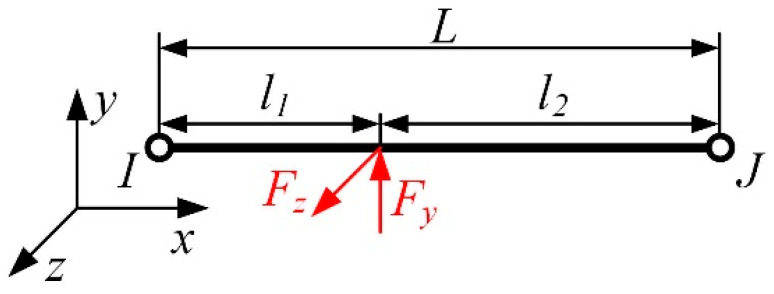
Equivalent load diagram of a beam element.

According to finite element theory, for a spatial six-degree-of-freedom beam element, as shown in Figure 2, *I* and *J* represent the two nodes of an element, *L* represents the length of the element, *F_y_* and *F_z_* represent the loads applied in the y and z directions, respectively, and *l*_1_ and *l*_2_ and represent the distances between the load application point and the *I* and *J* nodes, respectively. The displacement vector can be represented as [45]:

(1) $$U^{e}={\left[u_{1},v_{1},w_{1},\theta_{x1},\theta_{y1},\theta_{z1},u_{2},v_{2},w_{2},\theta_{x2},\theta_{y2},\theta_{z2}\right]}^{T}$$

where, $u_{1}$, $v_{1}$, and $w_{1}$ represent the displacements of node *I* along the three coordinate axes; $\theta_{x1}$, $\theta_{y1}$, and $\theta_{z1}$ represent the rotations of node *I* about the three coordinate axes; $u_{2}$, $v_{2}$, and $w_{2}$ represent the displacements of node *J* along the three coordinate axes; $\theta_{x2}$, $\theta_{y2}$, and $\theta_{z2}$ represent the rotations of node *J* about the three coordinate axes. The stiffness matrix of a spatial beam element considering shear effects can be represented as [46]

(2) $$K_{b}=\left[\begin{array}{cccc} K_{1} & K_{2}^{T} & -K_{1} & K_{2}^{T}\\ K_{2} & K_{3} & -K_{2} & K_{4}\\ -K_{1} & -K_{2}^{T} & K_{1} & -K_{2}^{T}\\ K_{2} & K_{4} & -K_{2} & K_{3} \end{array}\right]$$

where

(3) $$K_{1}=\left[\begin{array}{ccc} \frac{EA}{L} & 0 & 0\\ 0 & \frac{12EI_{z}}{L^{3}(1+\phi_{y})} & 0\\ 0 & 0 & \frac{12EI_{y}}{L^{3}(1+\phi_{z})} \end{array}\right],\;K_{2}=\left[\begin{array}{ccc} 0 & 0 & 0\\ 0 & 0 & \frac{-6EI_{y}}{L^{2}(1+\phi_{z})}\\ 0 & \frac{6EI_{z}}{L^{2}(1+\phi_{y})} & 0 \end{array}\right]\;K_{3}=\left[\begin{array}{ccc} \frac{GJ}{L} & 0 & 0\\ 0 & \frac{(4+\phi_{z})EI_{y}}{L(1+\phi_{z})} & 0\\ 0 & 0 & \frac{(4+\phi_{y})EI_{z}}{L(1+\phi_{y})} \end{array}\right],\;K_{4}=\left[\begin{array}{ccc} \frac{-GJ}{L} & 0 & 0\\ 0 & \frac{(2-\phi_{z})EI_{y}}{L(1+\phi_{z})} & 0\\ 0 & 0 & \frac{(2-\phi_{y})EI_{z}}{L(1+\phi_{y})} \end{array}\right]$$

In the equation, *E* represents the elastic modulus of the material; *A* represents the cross-sectional area of the beam; $I_{y}$ represents the moment of inertia about the *y*-axis of the cross-section; $I_{z}$ represents the moment of inertia about the *z*-axis of the cross-section; *L* represents the original length of the element; *G* represents the shear modulus; $\phi_{y}=\frac{12EI_{z}}{GA_{y}L^{2}}$, where $A_{y}$ represents the shear area in the *y*-axis direction; and $\phi_{z}=\frac{12EI_{y}}{GA_{z}L^{2}}$, where $A_{z}$ is the shear area in the *z*-axis direction. The mass matrix of the beam element considering shear effects is:

(4) $$M^{e}=\left[\begin{array}{cccc} M_{1} & M_{2}^{T} & M_{3} & M_{4}^{T}\\ M_{2} & M_{5} & -M_{4} & M_{6}\\ M_{3} & -M_{4}^{T} & M_{1} & -M_{2}^{T}\\ M_{4} & M_{6} & -M_{2} & M_{5} \end{array}\right]$$

where

(5) $$M_{1}=\left[\begin{array}{ccc} \frac{1}{3} & 0 & 0\\ 0 & A_{z} & 0\\ 0 & 0 & A_{y} \end{array}\right],\;M_{2}=\left[\begin{array}{ccc} 0 & 0 & 0\\ 0 & 0 & -C_{y}\\ 0 & C_{z} & 0 \end{array}\right],\;M_{3}=\left[\begin{array}{ccc} \frac{1}{6} & 0 & 0\\ 0 & B_{z} & 0\\ 0 & 0 & B_{y} \end{array}\right]$$

(6) $$M_{4}=\left[\begin{array}{ccc} 0 & 0 & 0\\ 0 & 0 & D_{y}\\ 0 & -D_{z} & 0 \end{array}\right],\;M_{5}=\left[\begin{array}{ccc} \frac{J_{x}A}{3} & 0 & 0\\ 0 & E_{y} & 0\\ 0 & 0 & E_{z} \end{array}\right],\;M_{6}=\left[\begin{array}{ccc} \frac{J_{x}A}{6} & 0 & 0\\ 0 & F_{y} & 0\\ 0 & 0 & F_{z} \end{array}\right]$$

As for the damping matrix of the beam element, it is calculated using Rayleigh damping, which simplifies to a linear combination of the mass matrix and the stiffness matrix, as shown below [47]

(7) C=αM+βK

In the equation, α, β can be calculated from the damping ratios of any two mode shapes and their corresponding natural frequencies.

(8) $$\left\{\begin{array}{l} \alpha =\frac{2(\zeta_{i}\omega_{j}-\zeta_{j}\omega_{i})}{\omega_{j}^{2}-\omega_{i}^{2}}\omega_{i}\omega_{j}\\ \beta =\frac{2(\zeta_{j}\omega_{j}-\zeta_{i}\omega_{i})}{\omega_{j}^{2}-\omega_{i}^{2}} \end{array}\right.$$

where $\omega_{i},\omega_{j}$ represents the natural frequency of the *i* and *j*th mode shape, and $\zeta_{i},\;\zeta_{j}$ represent the corresponding damping ratio.

When there are non-nodal loads acting on a spatial beam element, it is necessary to transfer the loads to the nodes, i.e., to convert the non-nodal loads into nodal loads. The concept of equivalent load is used, which ensures that the deformations and internal forces of the element are equal before and after the equivalence, as shown in Figure 2. For concentrated forces $F_{y}$ and $F_{z}$ acting on the element, according to structural mechanics, their equivalent nodal load is given by [48]:

(9) $$\left\{\begin{array}{l} F_{yI}=\frac{F_{y}l_{2}^{2}(3l_{1}+l_{2})}{L^{3}}\\ F_{yJ}=\frac{F_{y}l_{1}^{2}(3l_{2}+l_{1})}{L^{3}}\\ M_{zI}=\frac{F_{y}l_{1}l_{2}^{2}}{L^{2}}\\ M_{zJ}=-\frac{F_{y}l_{1}^{2}l_{2}}{L^{2}} \end{array}\right.,\left\{\begin{array}{l} F_{zI}=\frac{F_{z}l_{2}^{2}(3l_{1}+l_{2})}{L^{3}}\\ F_{zJ}=\frac{F_{z}l_{1}^{2}(3l_{2}+l_{1})}{L^{3}}\\ M_{yI}=-\frac{F_{z}l_{1}l_{2}^{2}}{L^{2}}\\ M_{yJ}=\frac{F_{z}l_{1}^{2}l_{2}}{L^{2}} \end{array}\right.$$

The element mass matrix, stiffness matrix, and damping matrix of a spatial beam element are derived from the local coordinate system of the element. These matrices can be transformed into the global coordinate system by using a transformation matrix **T**. By applying the transformation matrix, the stiffness matrix, mass matrix, and damping matrix in the element coordinate system can be converted to the corresponding matrices in the global coordinate system.

(10) $$\left\{\begin{array}{c} K^{G}=T^{T}K^{e}T\\ M^{G}=T^{T}M^{e}T\\ C^{G}=T^{T}C^{e}T \end{array}\right.$$

**Figure 3:**
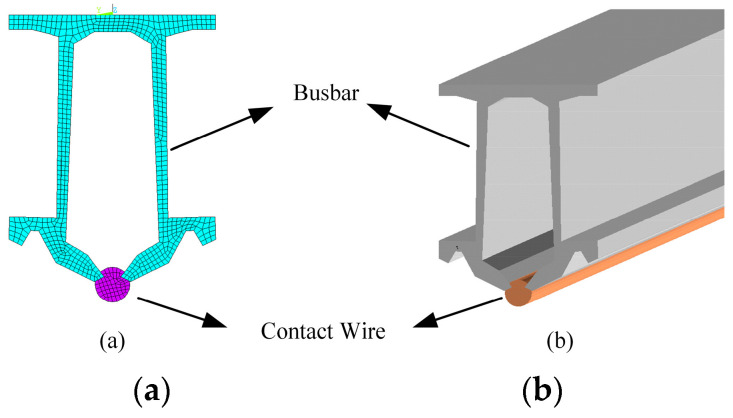
(**a**) Busbar-contact wire cross-section and (**b**) finite element model.

It is important to note that although the rigid overhead contact system’s busbar and contact wire move synchronously, they are made of different materials. Therefore, in the modelling process, it is necessary to consider the equivalent parameters of the composite cross-section. In this paper, the cross-sectional data for the busbar in the constructed rigid overhead contact system model can be found in Table 1, and its finite element model is shown in Figure 3. Figure 3a depicts the composite cross-section of the busbar and contact wire, while Figure 3b represents the finite element model of the busbar and contact wire. In the model, the busbar is made of aluminium alloy (blue portion), while the contact wire is made of copper–silver alloy (purple portion).

**Figure 4:**
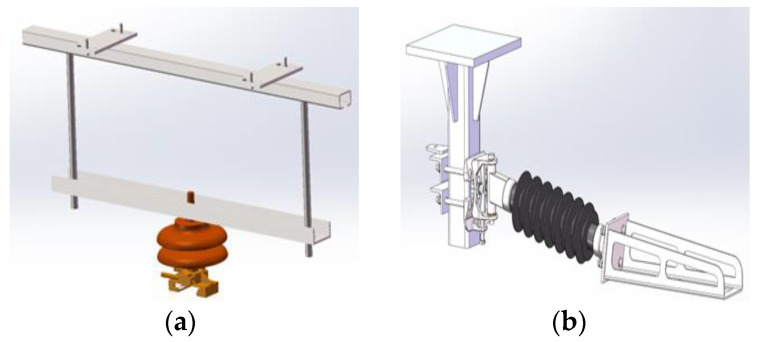
Rigid overhead contact system suspension structures: (**a**) gantry suspension structure; (**b**) cantilever suspension structure.

In the rigid overhead contact system, a suspension structure and a positioning line clamp are used to suspend and position the busbar. The suspension structure is usually fixedly installed at the top of the tunnel. The commonly used fixed structures are gantry and cantilever structures, as shown in Figure 4. The gantry suspension structure is simple but may cause the busbar to get stuck, so it is mainly used in rigid overhead contact systems of medium and low-speed urban rail transit. The cantilever suspension structure has good elasticity and can avoid the busbar from sticking by the rotational movement of the structure. It is suitable for longer anchor sections and is recommended for high-speed lines and mainline railways.

Compared to the busbar-contact wire structure, the suspension point has a very high stiffness. When the pantograph passes at high speed, it generates a certain impact on the pantograph head, resulting in significant fluctuations in contact force, which affects the stable contact and current collection of the pantograph. In order to reduce the abrupt change in local stiffness caused by the suspension structure in the rigid overhead contact system, it is common practice to increase the elasticity of the suspension point in high-speed sections to mitigate the impact on the pantograph.

**Figure 5:**
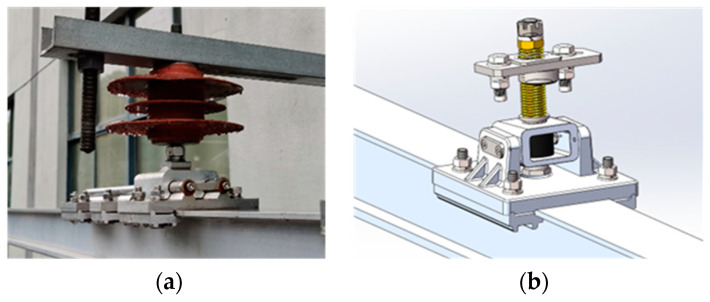
Rigid overhead contact system positioning line clamp: (**a**) rigid positioning line clamp; (**b**) elastic positioning line clamp.

Positioning line clamps can be classified into rigid positioning line clamps and elastic positioning line clamps based on their dynamic characteristics, as shown in Figure 5. The positioning line clamp is used in conjunction with the suspension system. Therefore, during modelling, the suspension structure and positioning line clamp are generally considered as a whole, and their dynamic characteristics are represented by equivalent stiffness, damping, and mass. The dynamic model can be simplified as a two-node mass-damping-spring element in the vertical direction, and the equation of motion for a single element can be expressed as:

(11) $$\left[\begin{array}{cc} m_{eq} & 0\\ 0 & m_{eq} \end{array}\right]\left[\begin{array}{c} {\ddot{u}}_{1}\\ {\ddot{u}}_{2} \end{array}\right]+\left[\begin{array}{cc} c_{eq} & 0\\ 0 & c_{eq} \end{array}\right]\left[\begin{array}{c} {\dot{u}}_{1}\\ {\dot{u}}_{2} \end{array}\right]+\left[\begin{array}{cc} k_{eq} & 0\\ 0 & k_{eq} \end{array}\right]\left[\begin{array}{c} u_{1}\\ u_{2} \end{array}\right]=\left[\begin{array}{c} f_{1}\\ f_{2} \end{array}\right]$$

where $m_{eq}$, $c_{eq}$, and $k_{eq}$ represent the equivalent mass, damping, and stiffness of the suspension structure and positioning line clamp, respectively. $u_{1}$ and $u_{2}$ represent the vertical displacements of the two nodes, while $f_{1}$ and $f_{2}$ represent the vertical loads applied to the two nodes. 

In addition to the general structures such as the busbar, contact wire, suspension structure, and positioning line clamp, the rigid overhead contact system also includes key components such as expansion joints, rigid-flexible transitions, intermediate joints, and centre anchor joints. The uneven distribution of stiffness and mass caused by these key components is an important factor leading to the deterioration of current collection quality in the high-speed operation of the pantograph. Therefore, the thesis constructs finite element models of the key components both at the system level of the rigid overhead contact system and at the component level.

**Figure 6:**
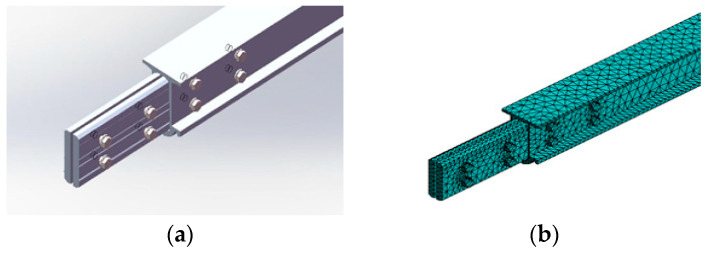
Rigid overhead contact system intermediate joint: (**a**) schematic diagram of the intermediate joint structure; (**b**) solid finite element model of the intermediate joint.

A single busbar is typically 12 m long, and the intermediate joint connects two busbars together. Figure 6 illustrates the schematic diagram of the intermediate joint. Inside the busbar at the intermediate joint, a fishplate is installed, which passes through the gap between the busbars and is secured by bolts, thus serving the purpose of connecting and fixing the two busbars. In the system-level model established in this paper, additional mass is added to simulate the intermediate joint, and the mass value is obtained by weighing the fishplate and assembled bolts. At the component level, the Solid45 element is used to discretize the fishplate, busbar, contact wire, bolts, and other components, and the contact relationship between these components is established.

With temperature changes, the rigid overhead contact system experiences thermal expansion and contraction phenomena in the busbars and contact wires. Accumulated thermal expansion and contraction in long anchor segments may lead to stress concentration in the busbars, affecting the smoothness of the rigid overhead contact system and even resulting in distortion and fracture failures. To address the expansion and contraction caused by environmental temperature changes, the rigid overhead contact system is divided into several independent segments of fixed lengths, with each segment called an “anchor segment”. Adjacent anchor segments are connected by anchor segment joints. The anchor segment joint consists of two parallel busbars, which maintain the continuity of the pantograph sliding track through alternating heights. It has a relatively simple structure, but unavoidable height deviations and changes in height slopes occur at the junctions of the two busbars, which may cause severe wear of the contact wire. The use of expansion joints not only buffers the axial expansion and contraction caused by temperature changes in the rigid overhead contact system but also provides a continuous connection between adjacent anchor segment busbars, ensuring smoother height transition and allowing the pantograph to pass at higher speeds. Expansion joints are crucial structures in the rigid overhead contact system, and it is necessary to analyse their stress distribution, explore their structural performance, and identify any potential weaknesses.

**Figure 7:**
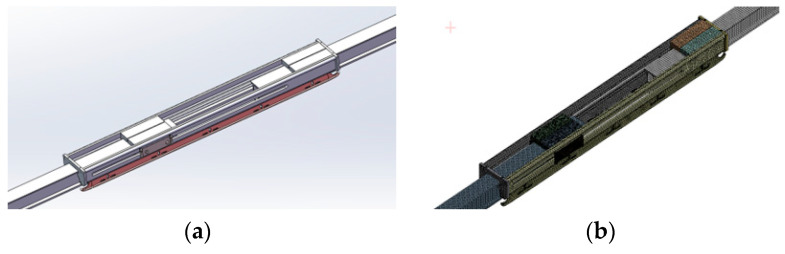
Expansion joint: (**a**) three-dimensional model; (**b**) solid finite element model.

According to the working conditions of the expansion joint, the method of additional beams is used to develop the model of the expansion joint in this paper. In the system model, according to the cross-sectional shape of the expansion joint, a U-groove cross-sectional beam is used to model the external additional sleeve of the expansion joint, which couples each degree of freedom with the two ends of the busbar, and only the degree of freedom of movement along the line direction is retained to represent the relative sliding movement between the busbar and the external sleeve. In the solid finite element model of the expansion joint, according to its three-dimensional dimensions, the solid45 element is used to discretize each part and assign the corresponding material parameters. Then, the face-to-face contact between the components is established to build the solid finite element model of the expansion joint, as shown in Figure 7.

## 3. Modelling of Expansion Joint

**Figure 8:**
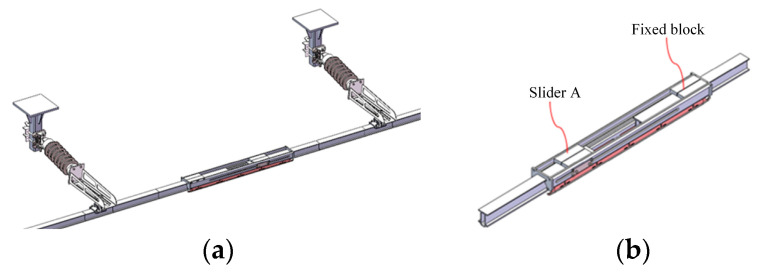
(**a**) Two-span rigid overhead contact system solid model. (**b**) Expansion joint solid model.

The expansion joint is an important component that connects different anchor sections of the high-speed rigid overhead contact system, as shown in Figure 8. It not only provides mechanical interconnection between different anchor sections but also enables electrical continuity. Its dynamic and static performance directly affects the current collection quality of the locomotive at the joint position.

**Figure 9:**
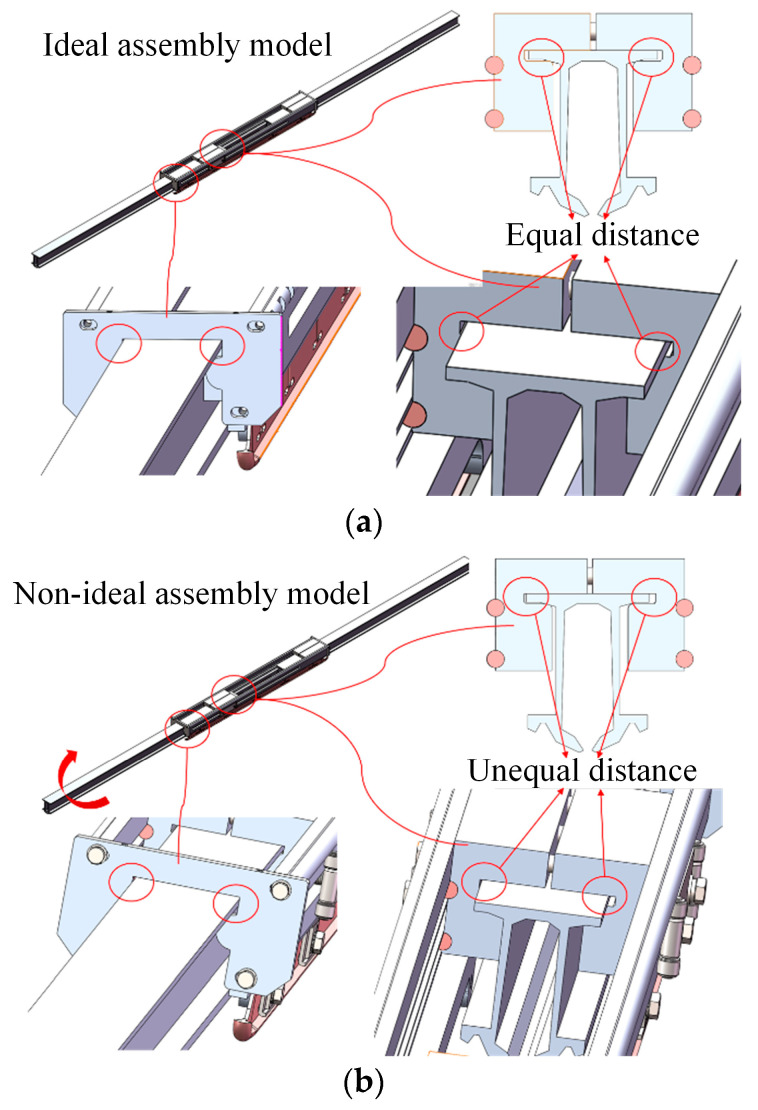
(**a**) Ideal assembly model of the expansion joint. (**b**) Deflected assembly model of the expansion joint.

In this section, a three-dimensional finite element model is constructed for the actual expansion joint. Due to the practical construction conditions, the assembly of the rigid overhead contact system is often imperfect. Therefore, this paper considers different assembly scenarios for the expansion joint, including:

(1) Ideal assembly condition for the expansion joint (without component assembly errors or line pull-out), as shown in Figure 9a, referred to as the “ideal assembly model of the expansion joint”;

(2) Actual assembly condition (caused by component assembly errors and line pull-out, etc.) resulting in the deflection of the expansion joint, as shown in Figure 9b, with the contact wire being inclined at a certain angle inside the expansion joint, referred to as the “deflected assembly model of the expansion joint”.

Based on the working characteristics of the expansion joint, this study simulates the dynamic behaviour of the expansion joint in the dynamic model of the rigid contact system by using the method of adding additional beams. In the system model, a U-shaped beam with a cross-sectional shape similar to the expansion joint is added externally to simulate the expansion joint. It couples with the two end current collectors, retaining only the degree of freedom for movement along the alignment direction to represent the relative sliding motion between the current collector and the external sleeve. In the solid finite element model of the expansion joint, a detailed solid model is established based on the three-dimensional dimensions of each component.

**Figure 10:**
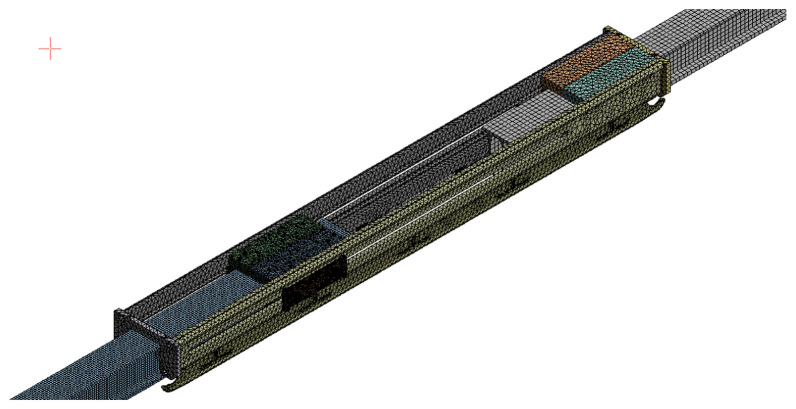
Finite element model of the expansion joint.

Here, we mainly introduce the modelling method for the solid finite element model. The structure of the expansion joint in the high-speed rigid overhead system is complex, consisting of different materials such as guide rail, sliding rod, current collector, slider, sliding plate, body, and bolts. Therefore, in the modelling process, not only the coupling relationship between different structures needs to be considered but also the material properties of different components. In this study, the selected materials for the guide rail and the sliding rod of the high-speed rigid overhead system expansion joint are copper alloy, while the current collector, slider, sliding plate, and body are made of aluminium alloy, and the bolts are made of structural steel. The material parameters are shown in Table 2. Each component is discretized using the solid45 element and assigned the corresponding material parameters. Contact surfaces are established between the components to create the solid finite element model of the expansion joint. The mesh division results of the three-dimensional finite element model for the expansion joint shown in Figure 8b are shown in Figure 10.

Considering the meshing of this model, while ensuring computational efficiency and accuracy, this study employs tetrahedral solid elements for the main body of the expansion joint, sliders (Slider A/Slider B), and sliding bars (Sliding A/Sliding B). The element size for sliders is set to 5 mm with a total of 39,173 elements, while for sliding bars, the element size is set to 3 mm with a total of 1976 elements. The remaining structures of the expansion joint are divided into elements of sizes ranging from 3 mm to 5 mm, resulting in a total of 238,899 elements for the entire expansion joint. The aim is to accurately capture the strain and stress concentration in these structures under temperature variations and simulate their sliding, friction, and compression characteristics. For other structures such as the cantilever, the entire busbar, and the contact line, a regular mesh size is used. The element size for the busbar is set to 15 mm, resulting in a total of 173,309 elements for the entire busbar in the model. This meshing approach ensures both computational accuracy and efficiency.

During the numerical calculation process, we first set the material properties of each substructure of the expansion joint based on their actual characteristics. The guide rail, sliding bar, and other components are assigned copper alloy material properties, while the busbar, slider, sliding plate, and main body are assigned aluminium alloy material properties. Structural steel material properties are assigned to bolts. Additionally, contact pairs that reflect the actual motion between different components are established, resulting in a total of 52 contact pairs among 31 bodies. These contact pairs adequately simulate the sliding, compression, and expansion processes between the substructures.

The main convergence method in this study involves calculating the norm of internal forces for all elements, referred to as the residual. The convergence tolerance is set at 0.001, and convergence is determined when the residual is smaller than the convergence tolerance. As for the boundary conditions, full constraints are applied to the cantilever device to simulate its fixed installation in the tunnel. The cantilever is connected to the busbar, the busbar is connected to the expansion joint, and the various substructures of the expansion joint are connected via surface-to-surface contact to represent the actual movement states of extension, sliding, and compression. These are the applied boundary conditions.

Regarding load application, the first step involves applying the gravitational field to check the initial configuration of the loaded model. Once verified, a global temperature field is applied to study the thermal expansion and contraction behaviour of the entire system. For the dynamic behaviour of the catenary-pantograph system, a pantograph model is incorporated, and the sliding contact of the pantograph is simulated by constraining the displacement of the pantograph head along the direction of the line. A penalty function method is used for contact treatment.

## 4. Analysis of Expansion Joint Stress Distribution

### 4.1. Stress Variation under Thermal Expansion and Contraction Effects (Normal Assembly)

**Figure 11:**
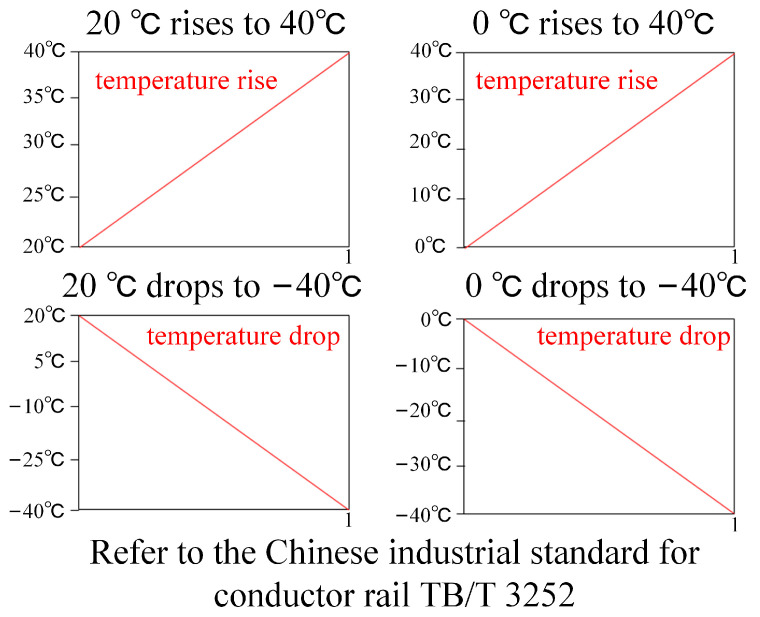
Four environmental temperature variation conditions (TB/T3252-2022).

According to [44], the suitable ambient temperature range for the busbar is −40 °C to 40 °C. Therefore, this study considers four temperature variations: normal temperature 20 °C to 40 °C temperature rise, 0 °C to 40 °C temperature rise, normal temperature 20 °C to −40 °C temperature drop, and 0 °C to −40 °C temperature drop, as shown in Figure 11.

**Figure 12:**
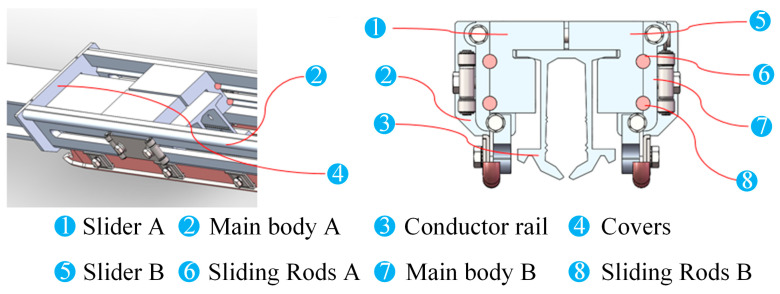
Solid model of the expansion joint.

**Figure 13:**
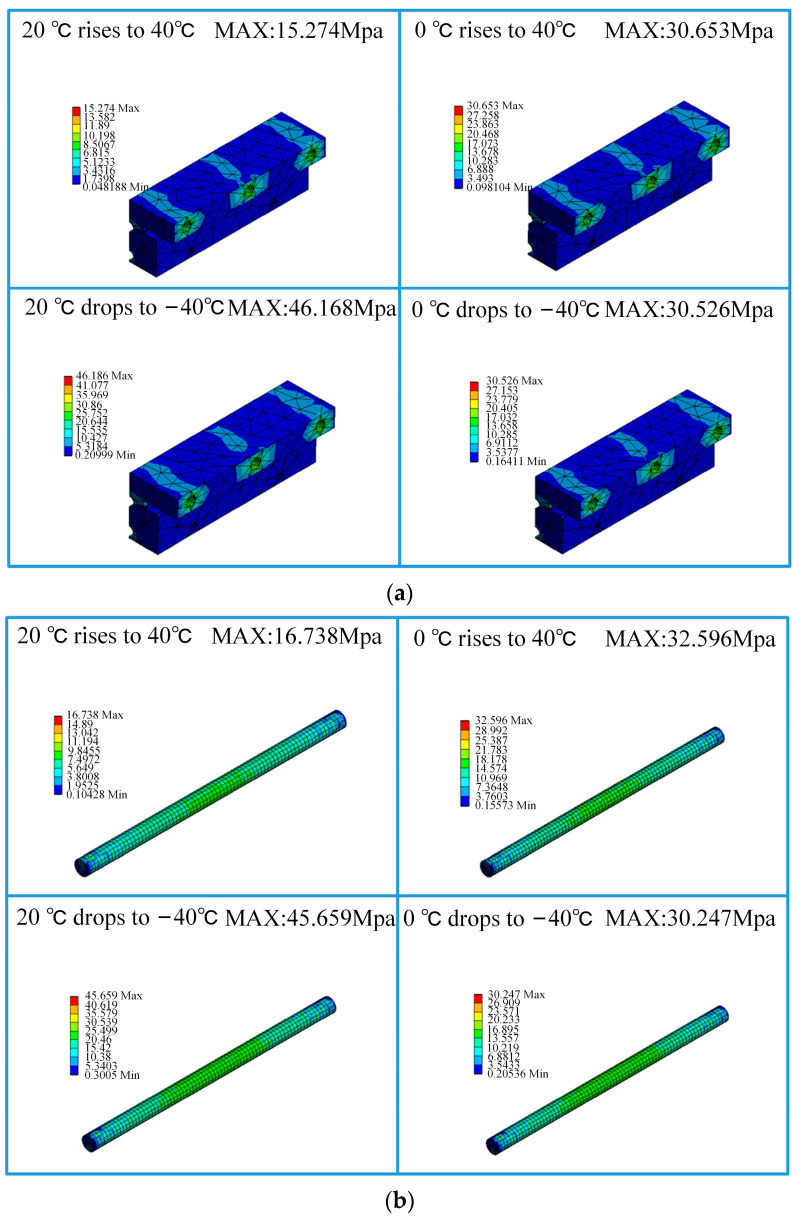
Stress contour maps: (**a**) Sliding Block B; (**b**) Sliding Rod A.

In this section, the components of the expansion joint are named as shown in Figure 12. The maximum simulated stress results of the main structures of the ideal assembled expansion joint model under the four temperature conditions, namely, normal temperature 20 °C to 40 °C temperature rise, 0 °C to 40 °C temperature rise, normal temperature 20 °C to −40 °C temperature drop, and 0 °C to −40 °C temperature drop, are presented in Table 3. According to the data in the table, it can be observed that under different temperature rise and drop conditions, the maximum stresses in the constructed expansion joint model occur at the Sliding Block B and the Sliding Rod A positions, as shown in the stress distribution contour maps in Figure 13. This is primarily due to the unique assembly form of the expansion joint. According to “TB/T3252-2022 Electrical Railway Contact Wire Busbar”, the tensile strength of aluminium alloy material should not be less than 215 MPa, and the yield strength should not be less than 160 MPa. Therefore, it can be concluded that in the ideal assembled expansion joint model without considering assembly errors and line pullout conditions, the maximum stresses in various structures are far below the permissible stress of the material, indicating a high level of safety.

### 4.2. Stress Variation under Thermal Expansion and Contraction Effects (Deflected Assembly)

**Figure 14:**
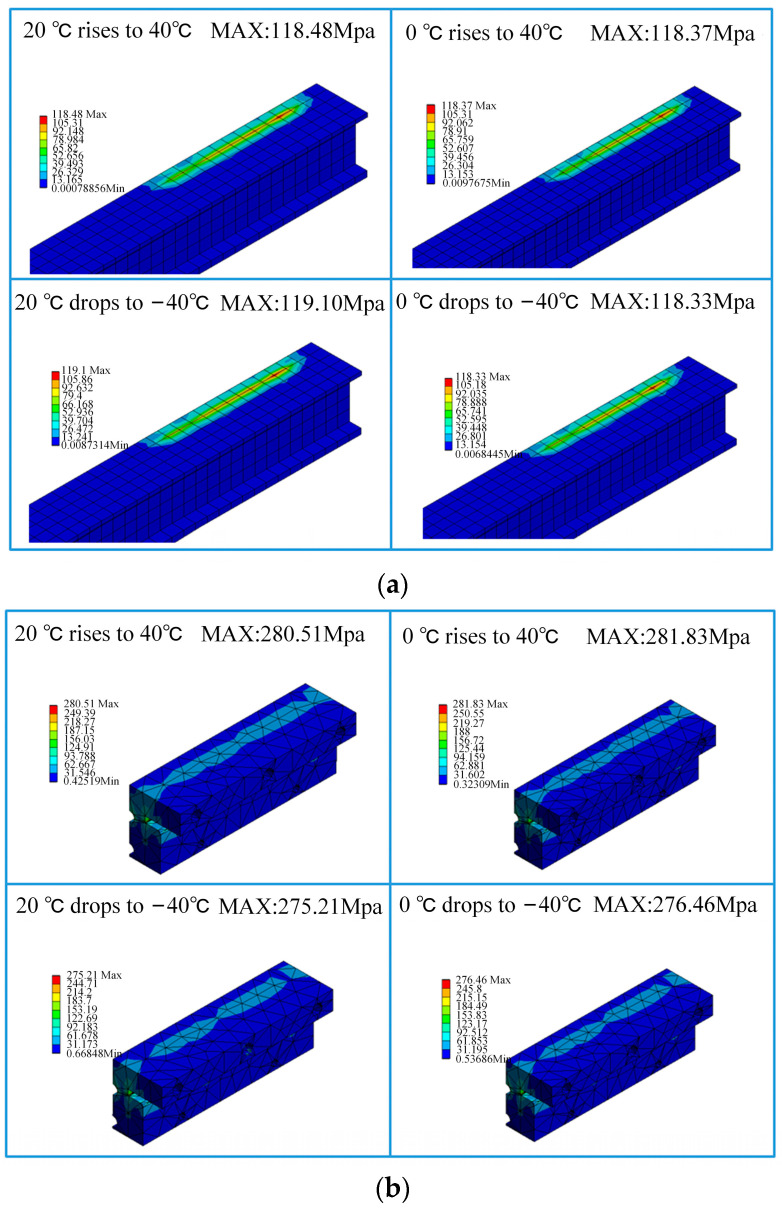

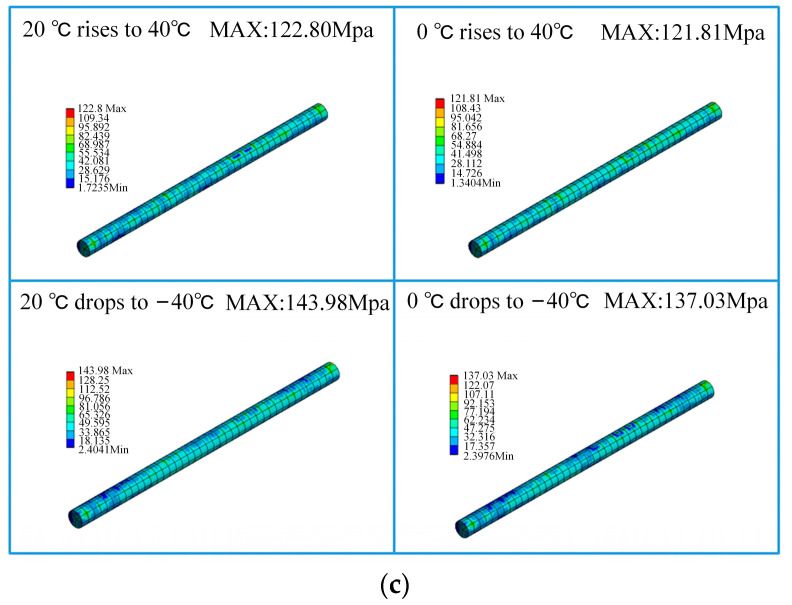
Stress distribution contour maps: (**a**) busbar stress; (**b**) Sliding Block B; (**c**) Sliding Rod A.

**Figure 15:**
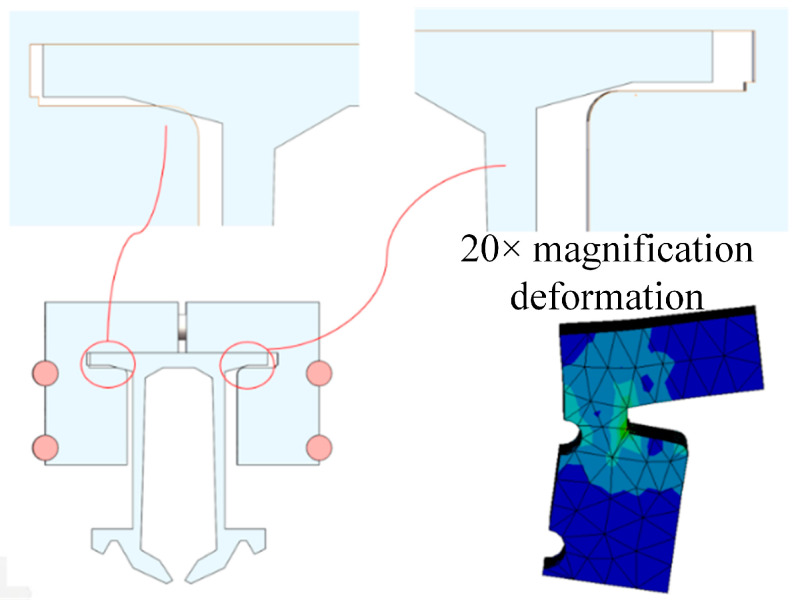
The process of stress concentration in the expansion joint is as follows.

The nomenclature of each structure of the expansion joint under deflected assembly conditions is the same as shown in Figure 12. The simulation results of the maximum stress in the main structures of the expansion joint under four temperature change conditions are shown in Table 4. From the data in the table, it can be observed that similar to the ideal assembly conditions, under deflected assembly conditions, the maximum stress in the expansion joint still occurs at the Sliding Block B and Sliding Rod A positions for different temperature rises and drops. Figure 14 shows the stress distribution contour maps of the three components. Unlike the ideal assembly conditions, under deflected assembly conditions, the busbar of the expansion joint also exhibits significant stress concentration, but it is still within the safe allowable range. According to relevant standards, the stress on Sliding Block B has exceeded the standard-specified stress, and the stress on Sliding Rod A and the busbar is close to the stress limit. The excessive stress in these three structures is mainly caused by the compression of the busbar on the groove of Sliding Block B during deflected assembly, resulting in stress concentration in that area, as shown in Figure 4 and Figure 5. Figure 15 shows the process of stress concentration in the expansion joint. Additionally, there is a certain amount of stress generated due to the compression of Sliding Block B on Sliding Rod A.

### 4.3. Optimization of Expansion Joints under Thermal Expansion and Contraction Effects

Based on the above analysis, it is known that the assembly deflection caused by line pull-out and construction errors is the main factor leading to stress concentration and increased risk of fracture in the expansion joint. In this section, based on the finite element model of the expansion joint, factors such as different assembly deflection angles and geometric design of the expansion joint are considered to analyse the optimization measures for stress distribution of high-speed rigid overhead system expansion joints under thermal expansion and contraction effects in tunnels.

#### 4.3.1. Installation Deflection Angle of the Expansion Joint

The deflection of the rigid overhead system expansion joint is generally caused by installation errors. Currently, relevant standards only specify the allowable horizontal height error of adjacent contact wires at the joint positions of the rigid overhead system. However, there are no specific regulations for the installation deflection angle of the expansion joint. In this section, based on the deflected assembly model of the constructed rigid overhead system expansion joint, the deflection angle of the expansion joint is adjusted to explore the influence of the deflection angle on the stress distribution and service reliability of the expansion joint. After reducing the installation deflection angle of the busbar, the simulated maximum stress results of the model are shown in Table 5.

**Figure 16:**
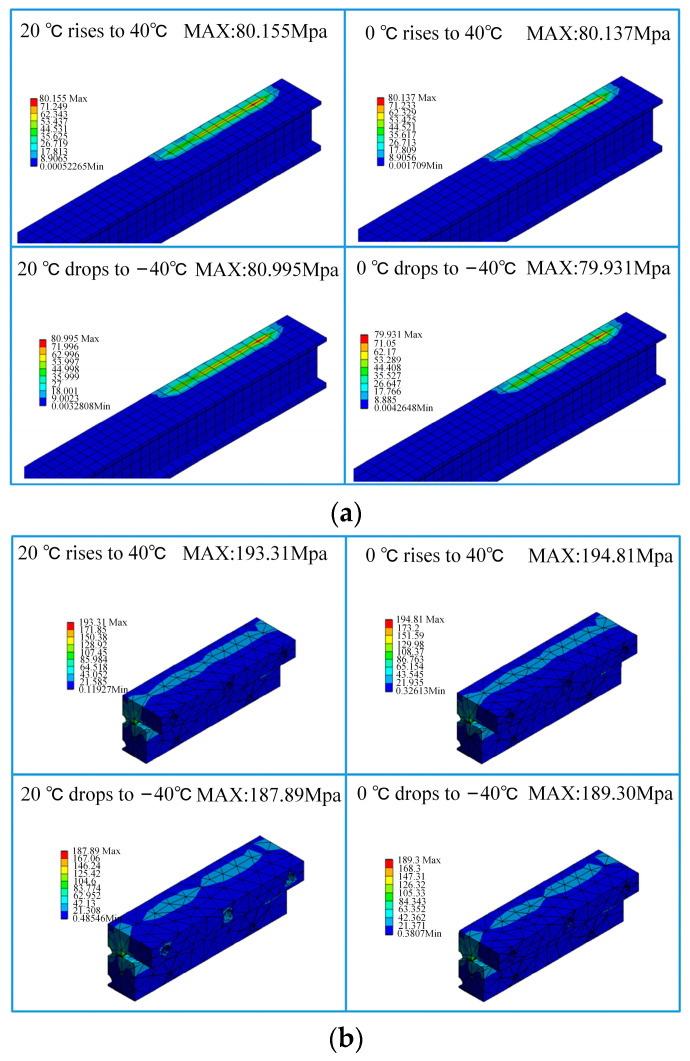

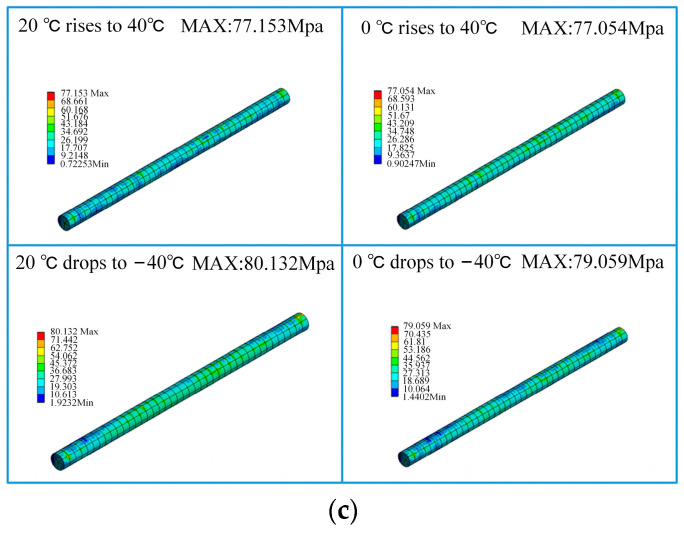
Stress distribution results: (**a**) conductor stress; (**b**) Slider B; (**c**) Slider A.

From the data in the table, it can be seen that after changing the deflection angle of the expansion joint, the stress concentration in the busbar, Sliding Block B, and Sliding Rod A is significantly improved, as shown in Figure 16. Among them, the stress concentration in Sliding Block B is reduced from a maximum of 281.83 MPa to 197.21 MPa, although it is still at a relatively high level. However, compared to the original assembly angle, the expansion joint as a whole under the condition of small deflection angle assembly meets the material safety requirements. However, as the stress is already lower than the ultimate tensile strength, long-term service can easily lead to local plastic deformation of the expansion joint, which may cause failures such as fractures and malfunctions. Therefore, during the construction and installation process of the joint positions in the rigid overhead system, the deflection angle of the expansion joint should be controlled as much as possible to improve the service life of the components.

#### 4.3.2. Geometric Design Optimization of Expansion Joint

**Figure 17:**
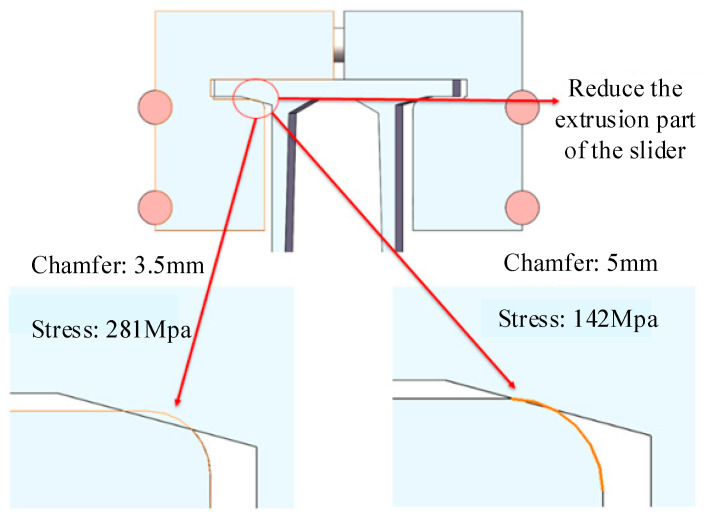
Expansion joint design improvement.

As mentioned in the previous section, the main reason for stress concentration at the position of Slider B in the deflection assembly condition is the abnormal compression at the groove of Slider B. Therefore, in addition to controlling the deflection angle of the expansion joint during construction and installation, optimizing the geometric design of the expansion joint to reduce the degree of compression at the groove position is also an effective solution for stress optimization. In this section, an appropriate improvement is made to the slider of the expansion joint by increasing the chamfer at the groove position from the initial 3.5 mm to 5 mm, as shown in Figure 17. The simulation results show a significant reduction in stress concentration at Slider B from the initial 281 MPa to 142 Mpa, indicating a significant improvement in optimization. Furthermore, at this assembly deflection angle, the stress concentration of the expansion joint meets the yield strength requirements of the relevant materials, effectively avoiding plastic deformation during long-term operation.

## 5. Dynamics Performance with Expansion Joints

**Figure 18:**
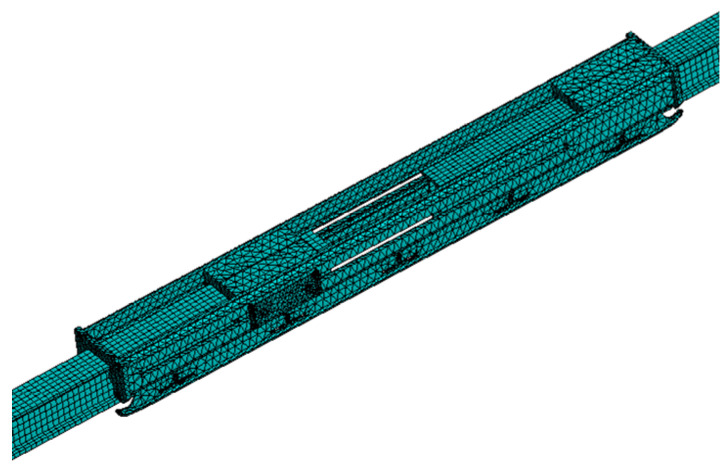
Finite element model of the rigid overhead system expansion joint.

In this section, based on the simulation model of the rigid overhead system and pantograph constructed in Section 2 and Section 3 of the paper, the influence of the structural parameters of the expansion joint on the current collection quality of the pantograph-overhead system is analysed. The overall performance of the pantograph-catenary interaction has been validated experimentally in the authors’ previous work [49]. The constructed rigid overhead system model has a length of 240 m and includes one anchor segment joint with an expansion joint transition. The finite element model of the expansion joint is shown in Figure 18. The main body of the expansion joint model is built using Solid185 solid elements, and the internal connections are established as a whole using the “tie” constraint. The external connections of the model are connected to the conductor rail and contact wire through the multi-point constraint (MPC) algorithm.

In the simulation of the rigid overhead system, beam elements with a certain mass are used to simulate the expansion joint, reflecting the influence of concentrated mass at the expansion joint on the pantograph through the mass to ensure calculation accuracy and improve efficiency. In the simulation model of the rigid overhead system, the concentrated mass of the expansion joint is calculated based on the finite element model shown in Figure 18. In this section, based on the conventional mass of the expansion joint (58 kg), four sets of expansion joint masses, namely 46.4 kg, 52.2 kg, 63.8 kg, and 69.6 kg are taken, with operating speeds set at 140 km/h, 160 km/h, and 180 km/h, to analyse the influence of the concentrated mass at the expansion joint on the current collection quality of the high-speed rigid overhead system.

**Figure 19:**
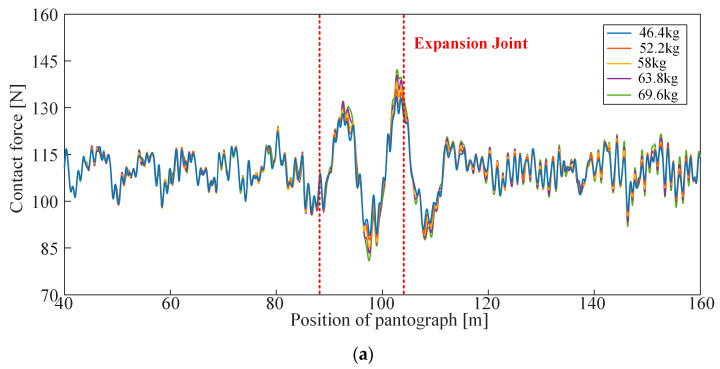

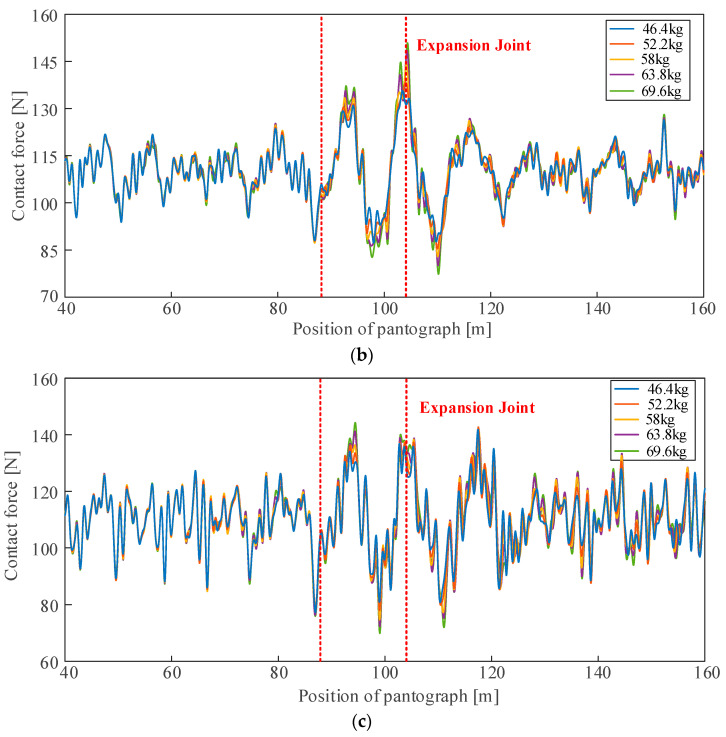
Time-domain curves of contact forces in the rigid overhead system for different expansion joint masses and speeds: (**a**) 140 km/h; (**b**) 160 km/h; (**c**) 180 km/h.

**Figure 20:**
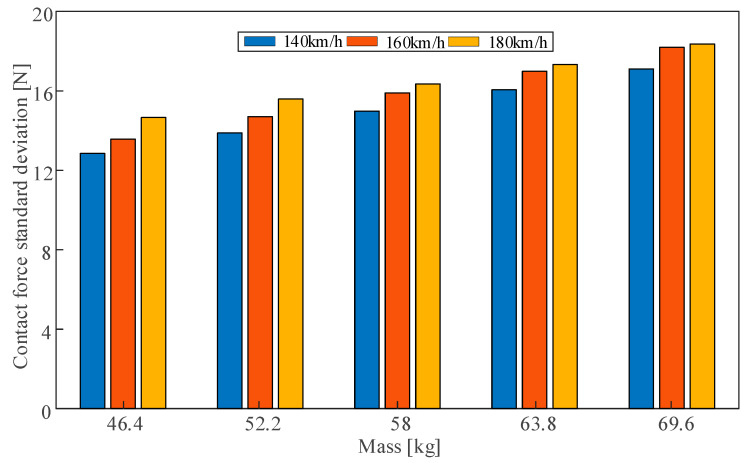
Standard deviation of contact force for different expansion joint masses and different speeds.

The simulation results are shown in Figure 19, and the numerical statistical results of the contact force on the pantograph are presented in Table 6. From Figure 19, it can be observed that the spans of the expansion joint and the adjacent spans are arranged according to the “6 m–4 m–6 m” rule, totalling 16 m. In other words, at the position of 90–106 m in the model, the pantograph enters the span before the expansion joint at 90 m, and the fluctuation of contact force starts to intensify. As the pantograph passes through the expansion joint at 96 m, the fluctuation of contact force becomes the most pronounced, with a significant increase in the standard deviation. When the pantograph passes the span after the expansion joint at 106 m, the fluctuation of contact force gradually decreases. Furthermore, when the speed is 140 km/h and 160 km/h, the fluctuation of contact force in the area of the expansion joint is much greater than in the general section. However, when the speed reaches 180 km/h, the fluctuation of contact force in the general section is similar to that in the area of the expansion joint. Additionally, it can be observed that the mass of the expansion joint has a significant impact on the contact force. Taking Figure 19a as an example, with the increase in the mass of the expansion joint, the contact force in the area of the expansion joint deteriorates significantly, with an increase in both the maximum value and the standard deviation of the contact force, while the minimum value of the contact force shows a decreasing trend. Therefore, the influence of the expansion joint on the dynamic characteristics of the rigid overhead system is mainly manifested in the local stiffness caused by mass concentration. Reducing the mass can effectively reduce the impact of the expansion joint on the flow quality of the rigid overhead-pantograph coupled system. Figure 20 specifically shows the standard deviation of the contact force. From the data in the table, it can be observed that as the mass of the expansion joint increases, the contact pressure on the pantograph in the rigid overhead system deteriorates significantly, with increased maximum contact pressure and standard deviation, while the minimum contact pressure shows a decreasing trend. Due to the installation method, there is no positioning line clamp above the expansion joint in the rigid overhead system. Therefore, its impact on the rigid overhead system mainly lies in the local hard point of the overhead system caused by the concentrated mass. Thus, reducing its mass can effectively reduce the negative impact of the expansion joint on the current collection quality of the pantograph-overhead system.

## 6. Conclusions

This research focuses on the numerical modelling of a rigid overhead system, specifically the dynamic characteristics and behaviour of its components. Suspension structures and positioning line clamps are employed in the rigid overhead system to suspend and position the busbar, and their dynamic characteristics are represented by equivalent stiffness, damping, and mass. Key components such as expansion joints, rigid-flexible transitions, intermediate joints, and centre anchor joints are also included in the modelling. The expansion joint, in particular, is crucial for connecting different anchor sections and ensuring mechanical interconnection and electrical continuity. Finite element models are constructed for the expansion joint under different assembly conditions, and the dynamic behaviour is simulated using additional beams. The solid finite element model of the expansion joint considers the complex structure and material properties of its components. The modelling process involves establishing contact surfaces between components and assigning material parameters.

The main conclusion from the analysis is that the maximum stresses in the constructed expansion joint model, under different temperature conditions and normal/deflected assembly conditions, are within the permissible stress limits of the material, indicating a high level of safety. However, there are areas of stress concentration, particularly at the Sliding Block B and Sliding Rod A positions. The stress concentration is primarily caused by the unique assembly form of the expansion joint. The analysis suggests that optimizing the installation deflection angle and geometric design of the expansion joint can improve stress distribution and service reliability. Additionally, the concentrated mass at the expansion joint has a significant impact on the current collection quality of the pantograph-overhead system, and reducing the mass of the expansion joint can mitigate this negative impact.

## Figures and Tables

**Table 1:** Busbar cross-section.

Item	Cross-Sectional Area [mm^2^]	Linear Density [kg/m]	Inertia of Moment [m^4^]
Value	2322	7.3	3.76 × 10^−6^

**Table 2:** Primary material properties of expansion joints.

	Copper Alloy	Aluminium Alloy	Structural Steel.	Units
Density	8.3 × 10^−6^	2.77 × 10^−6^	7.85 × 10^−6^	kg/mm^3^
Elastic modulus	1.1 × 10^5^	7.1 × 10^4^	2 × 10^5^	MPa
Thermal conductivity	0.401	0.14862	0.0605	W/mm·°C
Specific heat	3.85 × 10^5^	8.75 × 10^5^	4.34 × 10^5^	mJ/kg·°C

**Table 3:** Maximum stress results of the main structures in the ideal assembled expansion joint model (unit: MPa).

	Components	Busbar	Sliding Block A	Sliding Block B	Sliding Rod A	Sliding Rod B	Body A	Body B	Cover Plate
Temperature									
20–40 °C		2.15	9.04	15.27	16.74	7.47	5.30	5.33	6.67
0–40 °C		5.24	18.19	30.65	32.60	14.91	10.18	10.33	7.76
20–40 °C		6.70	26.99	46.19	45.66	22.19	15.52	15.70	6.82
0–40 °C		4.62	17.84	30.53	30.25	14.78	10.18	10.95	7.64

**Table 4:** Maximum stress results in the main structures of the expansion joint under deflected assembly conditions (unit: MPa).

	Components	Busbar	Sliding Block A	Sliding Block B	Sliding Rod A	Sliding Rod B	Body A	Body B	Cover Plate
Temperature									
20–40 °C		118.48	9.53	280.51	122.8	16.14	9.94	10.99	14.81
0–40 °C		118.37	18.23	281.83	121.81	15.65	11.88	12.61	15.77
20–40 °C		119.10	27.48	275.21	143.98	23.65	15.19	23.21	10.33
0–40 °C		118.33	18.44	276.46	137.03	19.06	10.25	19.74	11.24

**Table 5:** Maximum stress results of the deflected model of the expansion joint (unit: MPa).

	Components	Deflection Angle (10°)			Deflection Angle (5°)		
Temperature		Busbar	Sliding Block B	Sliding Rod A	Busbar	Sliding Block B	Sliding Rod A
20 °C–40 °C		118.48	280.51	122.8	80.16	193.31	77.15
0 °C–40 °C		118.37	281.83	121.81	80.14	197.21	77.05
20 °C–40 °C		119.10	275.21	143.98	81.00	187.89	80.13
0 °C–40 °C		118.33	276.46	137.03	79.93	189.30	79.06

**Table 6:** Statistical values of contact pressure on the pantograph for different expansion joint masses and speeds.

Speed [km/h]	Expansion Joint Mass [kg]	Maximum Contact Force [N]	Average Contact Force *F*_m_ [N]	Minimum Contact Force [N]	Contact Force Standard Deviation *σ* (N)	0.3 *F*_m_-*σ* (N)
140	46.4	133.55	112.19	89.07	12.85	20.81
	52.2	135.91	112.35	87.63	13.88	19.82
	58	138.14	112.47	85.11	14.98	18.76
	63.8	140.39	112.62	83.36	16.06	17.73
	69.6	142.22	112.73	80.90	17.11	16.71
160	46.4	135.62	111.64	87.12	13.57	19.92
	52.2	138.26	111.66	88.05	14.71	18.79
	58	143.02	111.66	87.72	15.90	17.60
	63.8	143.67	111.64	86.21	16.99	16.50
	69.6	144.65	111.67	82.60	18.20	15.30
180	46.4	136.81	110.88	80.96	14.66	18.61
	52.2	137.21	110.83	78.01	15.59	17.66
	58	137.45	110.77	74.67	16.35	16.88
	63.8	141.13	110.63	72.23	17.32	15.87
	69.6	144.13	110.57	69.79	18.36	14.81

## Data Availability

Not applicable.
